# Real-Time Smartphone Monitoring Assessments as a Cognitive Biomarker of Alzheimer Disease: Protocol for a Development Study

**DOI:** 10.2196/93259

**Published:** 2026-07-03

**Authors:** Meaghan McKenna, John Torous, Eden Rozenblit, Matthew Flathers, Sean Ryan, Andrew Jin Soo Byun, Chun Lim

**Affiliations:** 1 Department of Neurology, Beth Israel Deaconess Medical Center, 330 Brookline Avenue, Boston, MA, 02215, United States, 1 617 667 2622; 2 Division of Digital Psychiatry, Beth Israel Deaconess Medical Center, Boston, MA, United States

**Keywords:** digital health, Alzheimer disease, mild cognitive impairment, smartphone, passive data, digital phenotyping, digital biomarkers, cognitive testing, monitoring

## Abstract

**Background:**

The diagnosis and monitoring of Alzheimer disease (AD) currently rely on clinician-administered, in-person, and cross-sectional pen-and-paper cognitive assessments. While clinically validated, these measures are time-intensive, infrequently administered, and limited in their ability to detect early, subtle, or short-term cognitive changes. Thus, more frequent, ecologically valid assessments are critical to improving sensitivity to early cognitive impairment and disease progression.

**Objective:**

This study aims to develop and pilot a smartphone-based assessment battery that combines active cognitive assessments with passive smartphone sensor data (eg, steps, sleep) and survey data to identify and longitudinally characterize cognitive impairment associated with AD.

**Methods:**

We developed a suite of digitized versions of standard cognitive tests alongside novel, game-based cognitive tests within the mindLAMP platform. Uniquely, these tests integrate into the platform’s mobile survey and digital phenotyping capabilities to produce a comprehensive assessment tool capable of simultaneously tracking self-reported, behavioral, and cognitive symptoms in real time. These tools were unified within the Smartphone Monitoring Assessment in Real Time–Alzheimer’s framework. Across a 6-month pilot study involving individuals with mild cognitive impairment or mild AD, we will examine the feasibility, acceptability, and longitudinal adherence to these assessments. We will compare digital cognitive and passive data streams against standard clinical assessments to evaluate their usefulness in detecting cognitive impairment and change over time.

**Results:**

Recruitment began in April of 2025. As of February 2026, 13 participants with mild cognitive impairment or AD (mean age 72.8, SD 6.5 y, 8 male) and 12 controls (mean age 71.6, SD 7.8 y, 6 male) have been enrolled; recruitment is ongoing. Preliminary analyses on participant compliance, passive data, and variations in game scores are in progress. Data analysis is expected to be completed by mid-2026, and we anticipate results to be published in 2027. This study is funded by the a2 Pilot Awards, a subaward of funding given to the Trustees of the University of Pennsylvania under the a2 Collective, beginning in April 2024.

**Conclusions:**

Smartphone-based cognitive assessments, when combined with digital phenotyping, offer a scalable and ecologically valid approach to detecting and monitoring AD in real-world settings. This framework has the potential to enhance early detection, enable continuous monitoring, and support future machine learning–based automated identification of cognitive impairment, ultimately facilitating earlier and more personalized care.

## Introduction

In the United States, 7.2 million people have Alzheimer disease (AD) [1], a slowly progressive neurodegenerative disease with the deterioration from cognitively normal to dementia taking years to decades [2]. Screening tests such as the Montreal Cognitive Assessment (MoCA) [3] and the Mini-Mental Status Exam [4] have reasonable specificity and sensitivity for detecting cognitive impairments, but the time required to administer even such simple cognitive tests precludes their use as routine screening exams by primary care physicians [5], let alone using these tests frequently as a measure of disease progression. The development of more accurate, faster, inexpensive, and universal methods to both diagnose patients earlier in their disease progression and to better measure cognitive decline has spurred innovation in new online and digital assessments [6].

The hallmark of AD is memory loss, but invariably, cognitive impairment will progress, often representing a spectrum of nonuniform changes across multiple domains, including executive, visual-spatial, and language [7]. However, mild deficits in nonmemory domains may not be detected in the day-to-day life of mild cognitive impairment (MCI) or earlier stages of AD and are not recorded until they become more severe in later stages of AD [8]. While digital tests nowadays focus on the most well-known domain of memory impairment [9-12], there is a need for testing across multiple domains to achieve a full clinical picture.

While current brief clinical scales do offer rapid testing across multiple domains, they are underused and present limitations. Cognitive screeners, such as the MoCA or Mini-Mental Status Exam [3,4], cover several of these domains, but a full neuropsychological battery can take up to 4 hours [13]; requires an in-person administrator; and is limited by arbitrary scoring, floor or ceiling effects, and the inability to capture patterns of deficits in real-world cognitive functioning [14]. Computerized and tablet versions of neuropsychological tests allow for the gathering of more fine-grained response metrics not previously possible with traditional pen and paper tests, such as response time [15]. Tests can be made adaptive and can easily assess different areas of cognition [16]. With such tests allowing for self-administration and reduced testing time, as well as a wider variety of data available and automated scoring, computerized testing can alleviate many of the limitations associated with a full neuropsychological battery. However, there remain concomitant challenges with cognitive testing that have shown limits with test-retest variability and the practice effects of cognitive testing [17].

The development of new biomarkers [18-20] now allows us the opportunity to identify Alzheimer pathology years before clinical symptoms, creating a sizable new group of patients in need of longitudinal cognitive assessments, as treatment remains restricted to those with clinical symptoms [21,22].

The use of technology, such as smartphones, is especially suited for collecting behavioral and cognitive data that can be difficult to gather in an in-office visit, as most participants now own these devices and will quickly receive notifications for surveys and cognitive assessments. Smartphones extend the potential of computerized assessments by enabling longitudinal data capture alongside novel behavioral data [23,24].

While many smartphone apps can offer cognitive tests [25], few also record behavior, which can be captured using smartphone digital phenotyping (eg, wandering behavior and sleep patterns) to identify at-risk patients. Likewise, while other apps have used digital phenotyping data to monitor behavior in MCI or mild AD, without cognitive testing, these have demonstrated limited use [26] in identifying behavioral biomarkers. While thousands of apps can collect self-reported symptoms derived from AD symptom questionnaires [27,28] and there is strong evidence that these reports correlate well with other diagnostic measures for AD [29] and are a leading predictor of cognitive trajectories [27], there have been few efforts to combine them with mobile cognitive and digital phenotyping behavioral data for a full assessment of deficits in MCI or mild AD.

To address this gap in combining passive sensing of behavior, digital testing of cognition, and self-reports of cognition and behavior, our team extended and adapted the digital clinical research platform [30] previously deployed in studies focusing on behavior or cognition in mental health conditions for innovative research in AD. Given that smartphone cognitive assessments have already been shown to be well accepted by patients with mild AD [30-34], this protocol focuses on assessing real-world use cases and results. Using advanced analytical tools and machine learning pipelines openly shared on GitHub [35], we have developed the Smartphone Monitoring Assessment in Real Time–Alzheimer’s (SMART-A) paradigm to combine several cognitive games for use in the home environment to continuously monitor progression via traditional testing metrics, novel cognitive tasks, and their fine-grained task results, along with real-world information captured via smartphone sensors and with self-reports from questionnaires. Using machine-based computational analysis of the raw data from these tests, we can examine what measures most accurately predict overall cognitive status, both for screening and monitoring purposes. As these tests can be self-administered at home, the detection of early symptoms, and thus earlier intervention, is more feasible as well. The innovation of the SMART-A paradigm involves leveraging the capability of smartphones to assess both active and passive data to capture real-time ecological momentary assessment, behavioral data, and surveys, in addition to the data collected via cognitive assessments. In this paper, we document the development of cognitive games hosted on the app and describe the piloting of the SMART-A paradigm in a clinical population.

## Methods

### MindLAMP App

SMART-A extends the open-source mindLAMP app developed by the Division of Digital Psychiatry at Beth Israel Deaconess Medical Center (BIDMC) [30]. MindLAMP is an adaptable platform for biomedical research. The platform is acceptable and feasible for use in older adults and patients with AD [36]. It includes a study portal, a smartphone app (available on both Apple and Android), a database, data analysis, and modeling tools [37]. With special research entitlements, mindLAMP2 can capture Apple HealthKit and Google Health Connect data directly from smartphones, including accelerometer, gyroscope, screen time, geolocation, ambient light, call/text logs, and phone charging time in a safe, ethical, and patient-centric manner (see the “Ethical Considerations” section), and transform this raw data into clinically meaningful constructs such as sleep, mobility, and behavioral patterns through custom pipelines [38,39]. In addition, mindLAMP supports survey or ecological momentary assessment data and currently contains several cognitive tests related to spatial span tasks and visual identification tasks, but also the ability to create new cognitive tasks.

### Cognitive Games

#### Overview

On a separate platform, clinicians at the Cognitive Neurology Unit at BIDMC and Harvard Medical School have created cognitive tasks for measuring cognitive impairment in patients with MCI or AD [40]. These games have been adapted to the mindLAMP2 platform. These tasks center on 4 main cognitive domains impacted by AD: Executive Function [41,42], Memory [43,44], Language [44-46], and Visuospatial [8,47].

#### Executive

##### Digit Span

**Figure 1.**
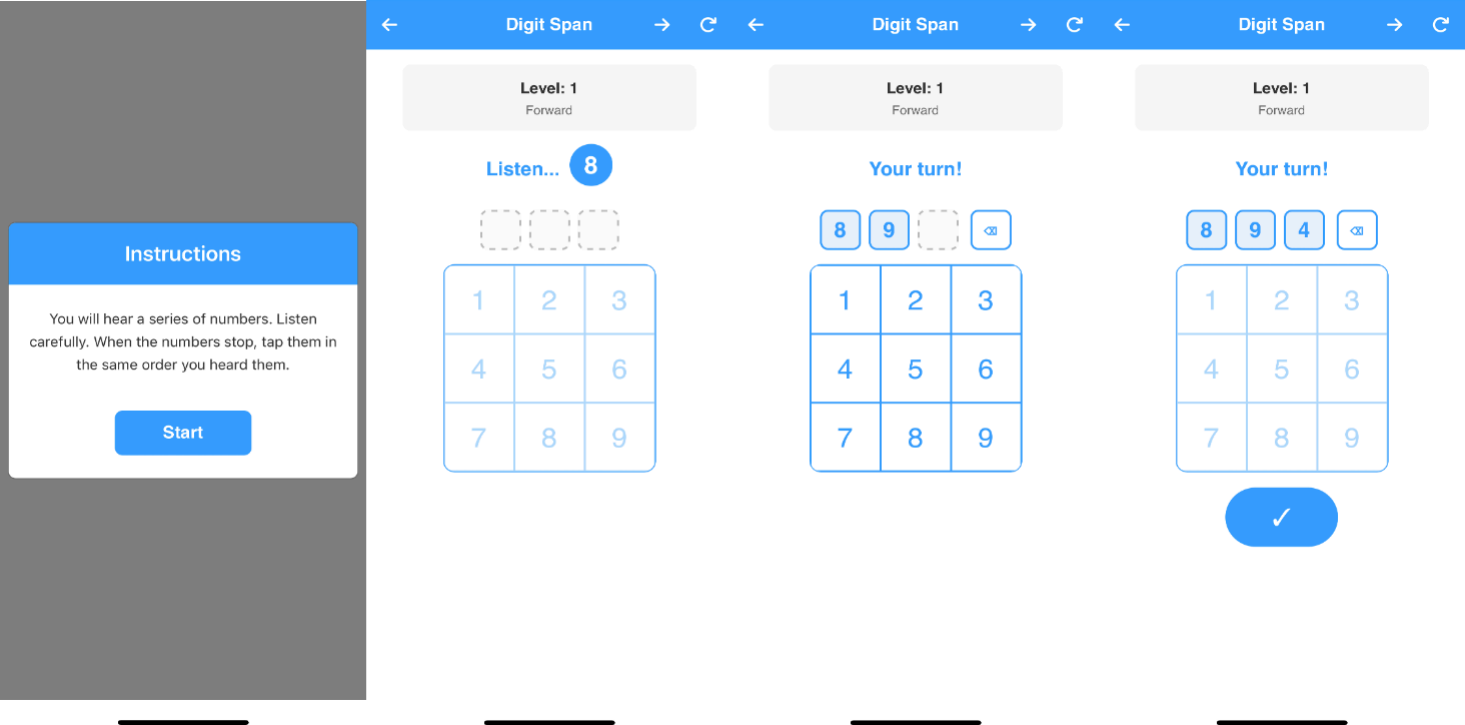
Screenshots from Digit Span task on mindLAMP.

This task is the same as the Digit Span Forwards or Backwards completed during many standard neuropsychology assessments [48]. A sequence of numbers (0‐10) is read aloud by an automated voice and shown one by one on the screen, and participants are asked to repeat the sequence by tapping the numbers on a phone screen, either in the forwards order or the backwards order. This is an adaptive game, meaning that the number of digits in the sequence will change depending on whether the participant got the previous sequence correct (Figure 1).

##### Touch Trails B

**Figure 2.**
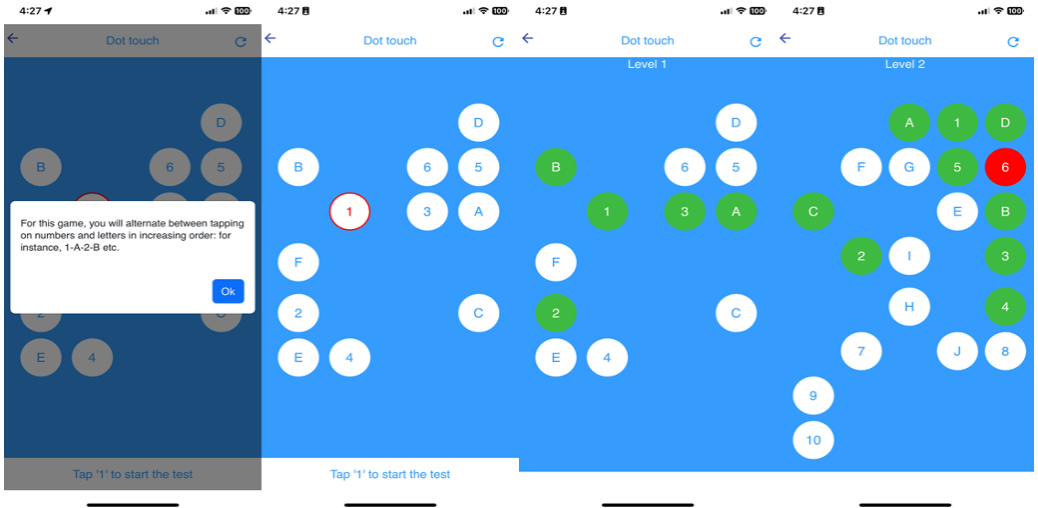
Screenshots from Trails B task on mindLAMP.

Adapted from the standard Trails Making Test [49,50], Touch Trails B (Figure 2) presents the user with an array of dots containing numbers and letters randomly placed on the screen, with the same amount of numbers (eg, 1‐6) and letters (eg, A-F), all customizable. The user is asked to select the dots in ascending order, tapping numbers and letters in alternation. Under the SMART-A paradigm, this version of Trails B contains 3 rounds; the second and third rounds contain more numbers and letters than the first.

### Memory

#### Associative Memory Game

**Figure 3.**
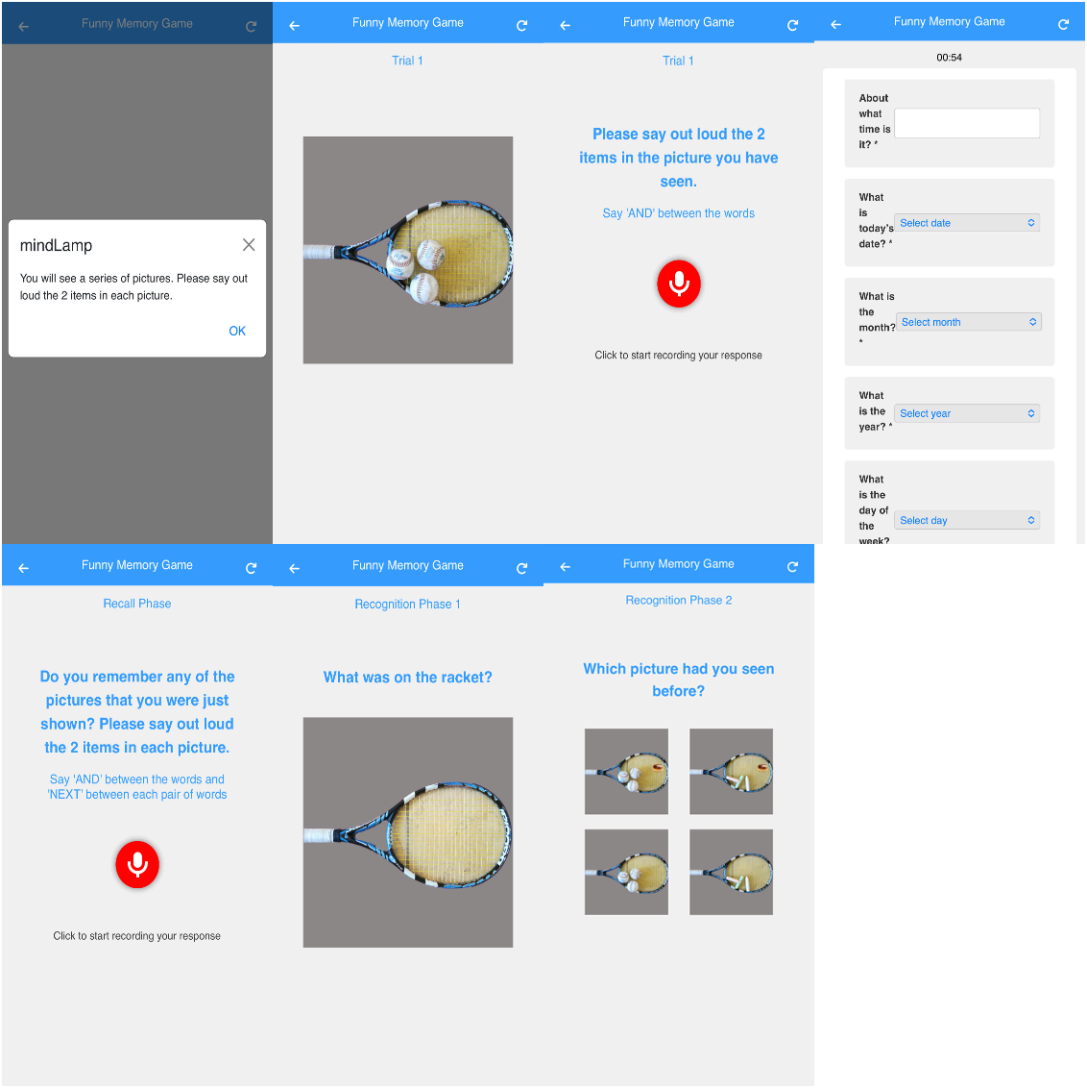
Screenshots from Associative Memory Test task on mindLAMP.

The Associative Memory Game (Figure 3), derived from visual association memory tests [51], asks participants to recall and recognize the combination between 2 unrelated items in the same image. The participant is briefly shown 6 images and asked to encode the objects by saying the objects out loud. After a customizable delay period (with orientation questions), participants are tested on free recall, cued recall, and multiple-choice recognition.

#### TicTacToe

**Figure 4.**
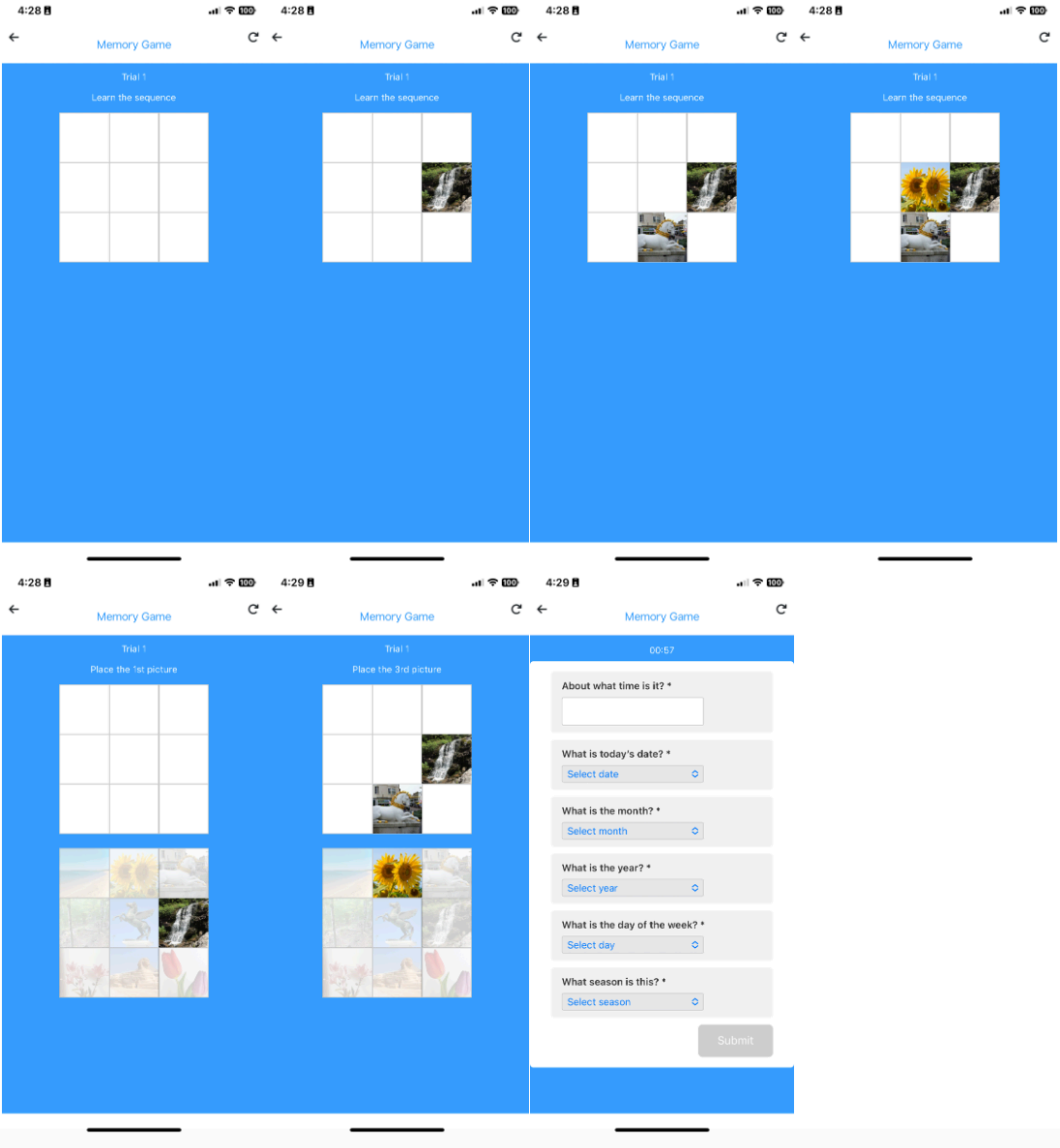
Screenshots from TicTacToe on mindLAMP 2 app.

TicTacToe (Figure 4) is a simple memory task involving visual, spatial, and temporal associative memory. A short sequence of photos from predetermined categories appears at random places on a grid. Participants are asked to identify both the order and location in which the photos appeared on the grid in a customizable number of learning phases, then asked to recreate the sequence following a short customizable delay period containing orientation questions.

### Language

**Figure 5.**
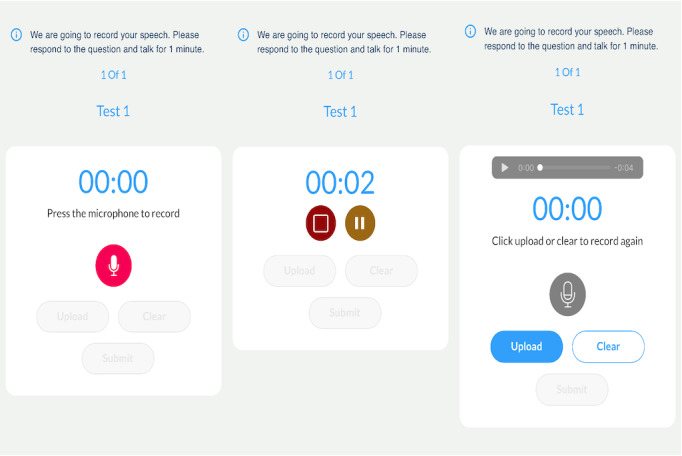
Screenshots of Speech Recording from mindLAMP 2 app.

In the Speech Recording task (Figure 5), participants will be given a prompt to respond to open-ended questions such as “describe what you had for breakfast this week.” Participants are asked to press the microphone to record their voice and verbally respond to the prompt for about a minute. The participant’s voice is recorded as an MP4 audio file.

### Visuospatial

**Figure 6.**
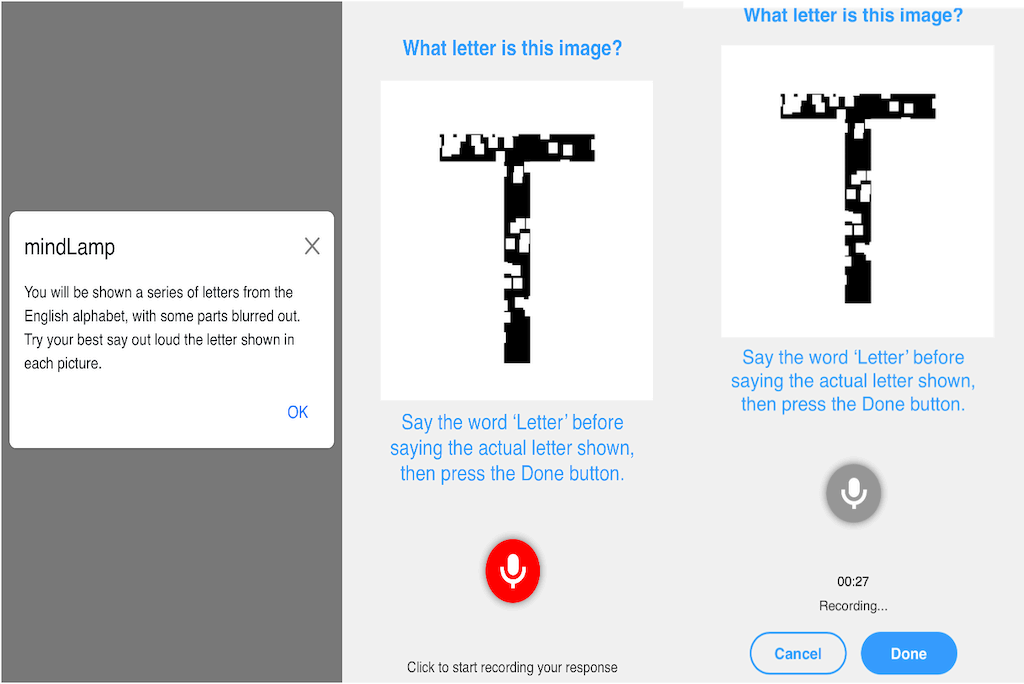
Screenshots of Fragmented Letters from mindLAMP 2 app.

During the Fragmented Letters task (Figure 6), the user is presented with letters of the alphabet written in black ink on a white background that have been slightly fragmented, causing some parts of the letters to blend in with the background [52]. Fragmentation is defined as the number of pixels in the letter that are white, out of the total number of pixels normally in the letter. In each round, the fragmentation percentage increases by 5%, and the game concludes after 10 trials. This game aims to assess both visuospatial and perceptual deficits.

### Survey Data

In addition to cognitive games, study participants are asked to complete brief surveys through the mindLAMP app. Orientation questionnaires drawn from the Orientation sections of the MoCA [3] and Clinical Dementia Rating (CDR) [53] are administered during the delay phase of TicTacToe and the Visual Association Memory Test. Drop-down menus are provided on the smartphone for questions such as “What month is it?,” and participants are asked to select the best answer.

Furthermore, participants are asked to complete a brief daily sleep survey and a longer weekly sleep survey. The daily sleep survey consists of 4 questions, inquiring about sleeping and rising time, and how long participants were awake (if at all) during the night. The weekly sleep survey provides several Likert scales for participants to rate how their sleep throughout the week affected their daytime activities.

### Digital Phenotyping

A unique facet of our approach is the use of smartphones to capture relevant and real-time behaviors via digital phenotyping. We will use the native features of mindLAMP to support digital phenotyping data collection to capture relevant information on behavioral domains pertinent to AD risk, including physical activity, sleep, and social interactions or isolation. While the assessment of these behaviors has traditionally been difficult, with smartphones, it is accessible and acceptable to patients with AD. Throughout the 6-month active data collection period, sensor data collection native to the mindLAMP 2 app [54] will also be activated, including GPS and accelerometer, communication events (anonymized call and text message logs), and phone and screen status. From these raw data streams, we will build relevant behavioral features in a transparent and reproducible manner that is not possible today with wearable data, where companies and the underlying algorithms can predefine features that can change at any time. The Cortex software package [55], developed as open-source software for use with mindLAMP data, will be used for initial behavioral feature creation.

### Study Protocol

We will recruit 15 patients evaluated by physicians in the Cognitive Neurology Unit of BIDMC with either MCI [56] or mild dementia [15] from AD. All participants with MCI or AD included in the study are diagnosed through neuropsychological testing with confirmatory biomarkers (eg, amyloid-positron emission tomography scan or p-tau 217). Fifteen cognitively normal controls, with no known history or diagnosis of cognitive impairment, will be recruited as well. Baseline evaluation will include the MoCA [3] and the CDR Global [53] score, as a staging measure for the MCI/AD group and a screening tool for the control group. Patients will run the SMART-A app on their smartphones for 6 months. During this period, we will capture passive data continuously and active data on a daily or weekly schedule (Table 1). As part of the Collective, data are entered into the a2 Collective Data Enclave, hosted by the Johns Hopkins Artificial Intelligence and Technology Collaboratory Data Integration and Quality Core. The a2 Collective aims to eventually archive the data in the National Archive of Computerized Data on Aging. All data sent to the Collective and entered into the Data Enclave will be completely deidentified and contain no protected health information. BIDMC and the University of Pennsylvania, the entity under which BIDMC was granted a subaward of the a2 Collective, will be sharing completely deidentified data with the NIH as well, per a previously agreed-upon protocol. Data will be available by request.

Over the course of 6 months, patients will complete each cognitive task weekly, a daily survey about sleep time and wake time, and a weekly survey about the quality of sleep. We anticipate collecting a total of 156 cognitive tasks, 180 daily sleep surveys, 26 sleep quality surveys, and 180 days of passive data over the course of the study from each participant. To optimize engagement, patients will be compensated a maximum of US $50 per month for engaging in cognitive tasks and surveys as well as for participating in the passive data collection. At the end of the 6-month study period, the baseline cognitive evaluations are repeated, and participants are given a survey (the mHealth App Usability Questionnaire [MAUQ]) on the usability of the app [57].

Participants with MCI or mild AD are offered optional continuation of the study for a maximum of 12 months. Our goal is for 5 to 10 participants from the AD group to continue this long-term data collection. When participants decide to end their participation, the final assessments completed at the 6-month end of study point (MoCA, CDR, and MAUQ) will be repeated.

**Table 1.** Weekly task rotation on mindLAMP app^a^.

Day of week	Sunday	Monday	Tuesday	Wednesday	Thursday	Friday	Saturday
Task 1	Daily mini-sleep survey	Daily mini-sleep survey	Daily mini-sleep survey	Daily mini-sleep survey	Daily mini-sleep survey	Daily mini-sleep survey	Daily mini-sleep survey
Task 2	Associative Memory Game	Weekly sleep survey	Digit Span	Trails B	TicTacToe	Fragmented Letters	Speech Recording

a Participants complete the Daily mini-sleep Survey each day and an additional game each day of the week. This schedule repeats for 6 months, or 26 weeks, giving us 26 datasets of each game, and approximately 180 mini-sleep surveys.

### Data Analysis

### Overview

The SMART-A study is designed as a longitudinal feasibility and proof-of-concept study of smartphone-based cognitive and behavioral monitoring in individuals with MCI or mild AD and cognitively normal controls. Given the modest sample size and the multimodal nature of the data collected, the analyses are organized into primary, secondary, and exploratory aims. This structure is intended to clearly distinguish feasibility-focused objectives from preliminary validity analyses and from more hypothesis-generating modeling work.

#### Primary Aims

The primary aims of this study are to evaluate the feasibility, adherence, passive data completeness, and usability of the SMART-A framework over the 6-month study period.

Feasibility will be assessed by examining rates of enrollment, retention, and study completion, as well as the ability of participants to engage with both active and passive components of the protocol in a home setting. This will include descriptive summaries of how many participants initiate the protocol, how long they remain engaged, and how many complete the end-of-study assessments.

Adherence will be assessed using completion rates for the scheduled cognitive tasks and surveys. We will summarize adherence at both the participant level and the task level, including the proportion of expected daily and weekly assessments completed, patterns of missed assessments over time, and variability in completion across different task types. We will also examine whether adherence changes over the course of the study and whether it differs between participants with cognitive impairment and controls.

Passive data completeness will be evaluated separately for each sensor stream, including GPS, accelerometer, phone and screen status, and communication event metadata. Because passive sensing data may be missing for technical or behavioral reasons, we will quantify the proportion of days with usable data for each stream and characterize the extent to which passive data are sufficiently complete to support feature generation. These analyses will help determine which passive sensing streams are most reliable and practical for use in this population.

Usability will be assessed primarily using participant responses on the MAUQ, along with qualitative feedback from participants and caregivers when available. We will summarize overall usability ratings and identify aspects of the platform that may require refinement for future studies involving older adults and individuals with cognitive impairment.

Together, these primary analyses are intended to determine whether the SMART-A paradigm can be implemented successfully in this population and whether the quality and completeness of the resulting data are sufficient to support future larger-scale validation studies.

#### Secondary Aims

The secondary aims of this study are to evaluate preliminary associations between smartphone-derived cognitive measures and standard clinical assessments, and to examine whether repeated digital assessments capture group differences and longitudinal patterns consistent with cognitive impairment.

First, we will examine the relationship between a prespecified digital cognitive composite and the baseline MoCA score. This digital composite will be constructed from a limited set of summary measures derived from the smartphone cognitive tasks, such as task accuracy, completion time, and response consistency, using a clinically informed and parsimonious approach. The purpose of this analysis is to assess preliminary convergent validity between the digital assessment battery and a widely used global cognitive screening measure. Because this is an early-stage study, these analyses are intended to estimate effect sizes and identify promising candidate features rather than to provide definitive validation.

Second, we will compare the digital cognitive composite between the MCI or mild AD group and the cognitively normal control group. This group comparison will provide an initial test of whether the smartphone-derived cognitive measures are sensitive to clinically meaningful differences in cognitive status. Given the sample size, these analyses will be interpreted cautiously, and emphasis will be placed on the magnitude and direction of group differences rather than on formal hypothesis testing alone.

Third, we will examine longitudinal changes in digital cognitive performance over time and whether those patterns differ by group. Repeated weekly digital assessments will allow us to assess within-person trajectories across the study period. These analyses will evaluate whether participants with MCI or mild AD show different patterns of performance, variability, or change over time relative to controls. Because each participant contributes multiple assessments, longitudinal analyses will account for the repeated-measures structure of the data and will use all available observations rather than requiring complete data at every time point.

These secondary analyses are intended to provide an initial assessment of whether smartphone-derived cognitive measures are meaningfully related to standard clinical measures and whether they can capture group-level and longitudinal differences relevant to cognitive impairment.

#### Exploratory Aims

The exploratory aims of this study are intended to generate hypotheses and identify promising directions for future work in digital cognitive biomarker development. These analyses will be considered preliminary and will not be interpreted as establishing a clinically validated prediction model.

One exploratory aim is to examine whether multimodal models that combine active cognitive task data, passive sensing data, and survey data provide stronger signals of cognitive status than any single data type alone. Because the number of potential features is large relative to the sample size, these analyses will use a reduced set of clinically motivated summary features and will prioritize interpretable and regularized modeling approaches. The purpose of these models is to identify candidate combinations of digital features that may warrant further study in larger cohorts.

A second exploratory aim is to develop a preliminary weighted digital scoring framework that integrates information across multiple smartphone-derived domains. This weighted score is intended as an early-stage summary measure of digital cognitive and behavioral functioning rather than as a finalized clinical algorithm. The goal is to determine whether a combined score may offer a more stable or informative marker than any individual task or passive feature alone.

A third exploratory aim is to examine speech recordings as a potential source of digital biomarkers. Speech data may contain clinically relevant information related to language production, fluency, timing, and content organization. In this study, speech analyses will focus on evaluating the feasibility of collecting usable recordings and on generating a limited set of candidate speech–derived features. Given the complexity of speech processing and the modest sample size, these analyses will be treated as hypothesis-generating.

A fourth exploratory aim is to examine whether digital measures collected over the study period are associated with end-of-study changes in clinical status, including changes in MoCA or CDR. Because standard clinical assessments are available only at baseline and end-of-study, and the study is not powered for definitive prediction of short-term change, these analyses will be interpreted cautiously. Their primary purpose is to identify preliminary signals suggesting which smartphone-derived measures may be most informative for future longitudinal prediction studies.

#### Analytic Approach

Analyses will proceed in a staged manner consistent with the above aims. Primary feasibility, adherence, passive-data completeness, and usability outcomes will be summarized descriptively using counts, proportions, means, SDs, medians, and CIs as appropriate. Visualizations of adherence and passive data availability over time will also be used to identify patterns of engagement and missingness.

For secondary aims, smartphone-derived cognitive features will first be reduced to a small number of prespecified summary measures in order to limit dimensionality and improve interpretability. These summaries will then be used to construct a digital cognitive composite. Associations between this composite and baseline MoCA will be examined using simple regression-based approaches, with adjustments for demographic factors such as age and education when appropriate. Group comparisons will similarly focus on prespecified summary measures and composite scores rather than large numbers of individual task features.

Longitudinal analyses will be used to evaluate changes in digital cognitive measures over time and differences in those trajectories between groups. These models will account for the correlation among repeated observations contributed by the same participant and will allow the inclusion of participants even when some weekly assessments are missed. Because this is a proof-of-concept study, these analyses will emphasize estimation, directionality, and consistency of effects.

Exploratory multimodal modeling will be performed using reduced feature sets and methods designed to limit overfitting. Model performance will be evaluated using out-of-sample procedures at the participant level so that repeated observations from the same individual do not contribute to both model training and evaluation. Any predictive findings from these exploratory analyses will be interpreted as preliminary and will require replication in an independent cohort before clinical conclusions can be drawn.

#### Missing Data

Missingness will be characterized separately for active and passive data streams. For cognitive tasks and surveys, we will describe the frequency and pattern of missed assessments by participant, task type, and study week. For passive sensing, we will distinguish between days with usable data, days with incomplete or poor-quality data, and days with no available sensor data. This distinction is important because passive data missingness may reflect technical issues, operating system restrictions, or participant behavior.

Primary and secondary longitudinal analyses will use all available data and will not require complete participation at every scheduled time point. Sensitivity analyses will be used where appropriate to assess whether findings are materially affected by data completeness thresholds or by differential missingness across participants or groups.

#### Interpretation

Given the modest sample size and proof-of-concept design, the study is not intended to provide definitive validation of a multimodal digital biomarker platform. Rather, its purpose is to determine whether this smartphone-based assessment framework is feasible and acceptable, estimate the variability and completeness of the resulting data, and identify the digital measures most promising for future larger-scale validation. The primary contribution of this study is therefore expected to be the generation of feasibility evidence, preliminary effect sizes, and candidate digital features that can guide subsequent confirmatory work.

### Power Analysis

This study is designed as a feasibility and proof-of-concept study rather than a definitively powered validation trial. Because reliable estimates of adherence, within-subject variability, sensor completeness, and effect sizes for smartphone-derived cognitive and behavioral features in MCI and mild AD are not yet established [58], this study is intended to generate the empirical parameters needed for formal powering of a subsequent larger study. The target sample of 15 participants with MCI or mild AD and 15 cognitively normal controls is expected to provide reasonable precision for estimating feasibility metrics and preliminary effect sizes, while supporting exploratory analyses of large between-group differences and longitudinal trajectories. For repeated-measures analyses, power will be evaluated using simulation based on the planned mixed-effects modeling framework, incorporating expected adherence, dropout, and within-subject correlation. All inferential findings from this study will therefore be interpreted as preliminary and hypothesis-generating.

### Ethical Considerations

We have received approval from the BIDMC Committee for Clinical Investigations, our institutional review board, for the outlined project (#2024P000931). Participants experiencing cognitive issues will be patients of the principal investigator and coinvestigators as well as patients of physicians at the Cognitive Neurology Unit. No patients will be approached unless they have given assent to be contacted about this study. We will recruit only participants who are able to consent to the testing, such as those with mild dementia (CDR of 1.0 or less), as determined by clinician rating prior to the screening process. All participants will be advised that they can withdraw from participation in this investigation at any time and that their participation in this research is completely voluntary.

For participants with dementia, a clinician who is familiar to the individual will discuss the study with the participant and their family member, review any questions they may have, and ask if they are willing to participate. Participants with dementia with a CDR of 1.0 or lower will be asked to sign the consent form. Although as a very general guideline, participants with dementia or a related disorder with a CDR of 2.0 or better might be considered capable of consent for the main study, our standard practice of “double consent” has been endorsed by the Alzheimer’s Association and is consistent with the suggestions of experts in the field and adheres to the “Points to Consider” published by the NIH Office of Extramural Research. In this context, having the participant and caregiver sign the informed consent for the participant makes perfect sense: it ensures that both the participant and caregiver are comfortable with the participation of the participant with memory impairment. Having both signatures on the consent form has several additional advantages. Allowing the participant with memory impairment to sign for themself along with the caregiver preserves dignity and is consistent with the humane clinical practice of addressing the patient and not just the caregiver in medical discussions.

Data security is one of the most important features of any mobile health study, and we have taken several safeguards to ensure data security in this pilot study. All personally identifiable information will be stored in REDCap (Research Electronic Data Capture) behind the BIDMC firewall, with access restricted to members of the research team. Only members of the research team will have access to the list of participants.

Passive data can potentially contain personally identifiable information, including GPS data and communication logs. The app’s GPS data provide enough detail to identify individual buildings or street addresses with some degree of confidence, although a fair amount of analysis would be required to transform a series of GPS coordinates into a home address for a participant. As our analysis will only look at changes in mobility patterns, no raw GPS data or specific locations will ever be investigated or reported. Call and text log data are data that tell when a phone call was made, or a text message was sent and how long that message was. The app does not collect any content from the phone call, so no data are recorded about what is said or what is typed. The app does record what phone number is called but encrypts this so that no one can determine the actual phone number that was communicated with. The app also records when the participant turns on or off the phone’s screen, when the participant turns off or reboots the phone, and when the participant plugs in or unplugs the phone. These data provide information on a participant’s typical phone usage and do not record any sensitive or identifiable information, only time stamps.

The app never records or stores any personally identifiable information. Every participant is assigned a randomly generated participant ID. For a participant to use the app, a study administrator must create a participant ID, and the user must register it and create a password. A participant ID can only be connected to one phone at a time. To upload data, a phone must have a valid username, password, and phone ID number.

All data on phones, on the server, and in-transit use industry-standard encryption techniques. The phone stores data locally only until they can be automatically sent to the secure server. The phone uses asymmetric encryption, meaning that even the phone cannot read its own data; data recorded on the phone can only be read on the server. Once Wi-Fi is available, the phone deletes any temporarily stored data and then sends them to the server in an encrypted manner. During registration, the device is provided with the public half of a 2048-bit RSA encryption key. With this key, the device can encrypt data, but only the server, which has the private key, can decrypt it. The RSA key is then used to encrypt a symmetric AES key for bulk encryption. These keys are generated as needed by the app, are not stored, and must be decrypted by the server before any data can be recovered. Data received by the BIDMC server are then re-encrypted with a master key provided for that study.

## Results

Recruitment began in April of 2025. To date, 13 participants with MCI or AD (mean age 72.8 y, 8 male) and 12 controls (mean age 71.6 y, 6 male) have been enrolled; recruitment is ongoing. Preliminary analyses on participant compliance, passive data, and variations in game scores are in progress. Data analysis is expected to be completed by mid-2026, and we anticipate results to be published in 2027. This study is funded by the a2 Pilot Awards, a subaward of funding given to the Trustees of the University of Pennsylvania under the a2 Collective, beginning in April 2024.

## Discussion

### Anticipated Findings

The pilot study described in this protocol aims to affirm that it is feasible for our population of participants with MCI and AD to complete the testing paradigm, and to identify which active and passive biomarkers have the most potential to differentiate between the MCI/AD and control groups. Larger-scale studies may contain some changes to the SMART-A paradigm based on our feasibility findings from the pilot study but will aim to confirm the strongest combination of active and passive biomarkers for AD.

We expect to find differences between the AD group and controls in passive data measures (eg, sleep and movement-related), as patients with AD have been documented to have sleep, socialization, and physical activity deficits [59-61]. While intraindividual variability is expected in all participants, we anticipate those with cognitive impairments to experience more fluctuations in their scores [62], and to exhibit less of a learning effect on the cognitive tasks [63]. Given that memory deficits are considered a hallmark of AD [7], we hypothesize that our cognitive games centered around the memory domain, the Associative Memory Game and TicTacToe, will be the most sensitive in differentiating between our cognitively impaired and cognitively unimpaired groups.

### Principal Findings

While longitudinal data collection for the pilot study is ongoing, the integration of these novel tests onto the mindLAMP platform highlights how feasible and scalable a digital clinical diagnostic program for AD can be. The feasibility of recruitment and interest in the study, indicated by recruitment numbers being nearly met in 8 months, suggest that this paradigm of smartphone-based multimodal data collection is acceptable and feasible in a cognitively impaired population. While concerns around digital literacy or privacy are valid, our study and others [64-66] indicate that older adults are very capable of using devices and willing to share personal data from their smartphones with the appropriate research safeguards in place. Participants who have completed the 6-month-long trial report that the mindLAMP 2 app was easy and enjoyable to use, and the weekly schedule of tasks was manageable to complete.

### Comparison to Prior Work

While other studies have investigated the use of digital measures for screening and monitoring of AD [23-26], our SMART-A paradigm is unique in combining multiple data streams from a real-world setting to assess several areas of functional and cognitive deficits. Passive data streams have not shown promising results as digital biomarkers for AD on their own [26], but we anticipate that, in combination with cognitive testing and survey reports, we will obtain a stronger signal than from any one data stream alone. As noted from other studies using the mindLAMP app, paradigms combining passive and active data streams in other clinical populations [67-69] are feasible and can provide at least preliminary results. The SMART-A paradigm proposed in this protocol has the potential to expand the applications of the mindLAMP app and to strengthen the currently available collection of digital biomarkers for AD.

### Strengths and Limitations

It is important to consider that this initial study is only a pilot, with a relatively low sample size of 15 MCI or AD and 15 controls, and a relatively homogeneous population. Most participants are highly educated (having earned a bachelor’s degree or higher), are generally comfortable with using technology, and are English speakers from the United States. Further work testing the feasibility and preliminary usefulness of the SMART-A paradigm in other groups will be needed to ensure its application to all groups. In addition, the sample size will need to be much larger to determine whether each digital biomarker can detect or predict cognitive impairment, instead of just a preliminary association. However, the results from the pilot study will inform future iterations of the SMART-A paradigm in larger-scale studies. Recruitment for this pilot study was limited to those with mild cognitive difficulties; while this was done to ensure participants had the capacity to understand the instructions of the cognitive games, further research will be needed to validate these tests as cognitive biomarkers in more advanced dementia populations. Many older adults use Android phones, and sometimes older phone models, as their primary device; however, the mindLAMP app is most compatible with iOS devices. Glitches or bugs are more common in those with Android devices, which can lead to more frustration and difficulty completing tasks than those with iOS devices. Due to the ever-evolving nature of technology, the mindLAMP platform is unable to support any software lower than Android 8, potentially posing a barrier to accessing the SMART-A paradigm.

### Future Directions

Our pilot study will establish preliminary data on the acceptance of the SMART-A paradigm in an MCI or AD population and on how the MCI/AD group differs on each measure from the control population. Further analysis of passive data, cognitive tests, and self-reported symptoms on a larger sample size than the pilot study presented here will allow us to optimize the SMART-A protocol to include only the measures that most accurately predict overall cognitive status, both for screening and monitoring purposes.

Our long-term goals are to develop a validated machine learning model to automatically detect cognitive and/or behavioral impairments and to incorporate this into an AI-grounded smartphone/tablet app, which can be used to identify and track patients with very early-stage AD while at home. This app will be used to assess different stages of cognitive impairment and detect clinically meaningful changes such as the transition from independence to supervision. Such an app will offer a range of uses as a diagnostic tool, for home monitoring, and to measure the efficacy of intervention trials. The use of ML models in analyzing the combination of passive and active data collected under this protocol is promising for the development of automatic cognitive monitoring over time, by using longitudinal patterns to make predictions. This allows us the ability to link these together and develop an adaptive version of the app that will actively pop-up cognitive tasks as soon as it detects clinically significant ecological data decline—often referred to as just-in-time adaptive interventions.

### Conclusions

In a world where older adults are more reliant on technology than ever before, we can capitalize on a patient’s own devices to glean information about their cognitive health. The innovation of the SMART-A paradigm involves leveraging the capability of smartphones to assess passive data to capture real-time ecological momentary assessments, behavioral data, and surveys, in addition to active data collected via cognitive assessments. Smartphone-based cognitive assessments, when combined with digital phenotyping, may offer a scalable and ecologically valid approach to detecting and monitoring AD in real-world settings. If the pilot study presented provides preliminary evidence of clinical usefulness in an MCI or AD population, future iterations of this framework have the potential to enhance early detection, enable continuous monitoring, and support future machine learning–based automated identification of cognitive impairment, ultimately facilitating earlier and more personalized care.

## Supplementary material

10.2196/93259 Peer Review Report 1 Peer review report 1 from a2 Collective and a2 Coordinating Center, National Institute on Aging (National Institutes of Health, USA).

10.2196/93259 Peer Review Report 2 Peer review report 2 from a2 Collective and a2 Coordinating Center, National Institute on Aging (National Institutes of Health, USA).

## Abbreviations

AD: Alzheimer disease
BIDMC: Beth Israel Deaconess Medical Center
CDR: Clinical Dementia Rating
MCI: mild cognitive impairment
MoCA: Montreal Cognitive Assessment
REDCap: Research Electronic Data Capture
SMART-A: Smartphone Monitoring Assessment in Real Time–Alzheimer’s
