# Supplementary material for: Real-Time Smartphone Monitoring Assessments as a Cognitive Biomarker of Alzheimer Disease: Protocol for a Development Study
Source: JMIR Res Protoc. 2026 Jul 3;15:e93259. doi: 10.2196/93259 (PMC13331333; doi:10.2196/93259)
Supplement: Peer Review Report 1 [file resprot-v15-e93259-s001.pdf]

Reviewer 1:

Significance: The project is highly significant due to low rates of detection of cognitive impairment and dementia among older adults and the need for feasible and acceptable approaches to increase detection.

Innovation: The project has several sources of innovation including the use of both passive and active data streams and the use of machine learning to identify markers of cognitive impairment.

Commercialization potential: Potential is high due to the use of the mindlamp application platform which could be adapted by other health systems.

Approach: The approach is appropriate and the methods are described in detail. For both Aims 1 and 2A, the hypotheses state that the proposed approach will "improve patient compliance" and "identify Alzheimer's disease patients with greater precision"; the comparison group for testing these hypotheses is unclear.

Investigators: The investigators have the skills and experience needed to complete the project.

Environment: The environment is outstanding and the project builds on existing BIDMC resources and work in this area.

Reviewer 2:

This project aims to develop a smartphone-based app (SMART-A) to capture data on AD patients, with the ultimately goal of being able to identify and track AD patients. The current project builds on a platform developed by the researchers for smartphone data collection and research, and aims to adapt and test it for the AD context. While the overall goal of the project is exciting and the researchers have experience in this domain, enthusiasm is dampened by weaknesses in the Approach.

Aim 1: There is a lot of description of the existing platform and its capabilities, but less information on what is actually being done in this Aim for the novel SMART-A platform, and how this will be done. Two sentences describe what will be adapted: presumably survey and memory questions as well as cognitive-based tasks. One notable strength here is the researcher’s attention to digital health engagement, which is an important (and oft-ignored) feature for downstream usability.

Aim 2: This Aim will identify the data that best detects and tracks cognitive impairment across two sub-aims. In Aim 2A, 15 AD and 15 controls will use the platform; in Aim 2B, a longitudinal study will be conducted with a subset of the AD patients that will also involve periodic in-clinic visits to assess cognitive and functional performance. This Aim lacked sufficient detail on study

procedures and justification: why were 15 patients selected (for each group) and will a month of data collection be sufficient to identify differences? How will healthy patients be recruited?

It is stated that subjects without smartphones will be loaned a smartphone in lieu of payment. While the gesture towards equity is recognized, the details need clarification - why is payment not provided to these individuals? One of the most significant issues with the provision of a second device in these types of studies is that they hardly get used (especially if subjects are accustomed to using a separate device for calls) thus there may be little passive data collection. This is especially a concern given that the smartphone will only be provided for a month. How will researchers ensure that subjects actually use the second device?

One of the main goals of Aim 1 is to develop tasks to collect cognitive data, but in Aim 2 there does not seem to be an assessment of these tasks (unless researchers are referring to these under the broad heading of active data which is a separate subheading in Aim 1 than cognitive data).

Details are lacking for Aim 2B and the budget justification: how many subjects expected to play how many games and for how long? Is the timeframe sufficient to capture significant changes in cognitive and functional scores?

Timeframes are unclear: all Aims are listed as occurring over the grant period with no further specifications.

Reviewer 3:

This is a really cool proposal that is proposing to modernize the clinical diagnosis of Alzheimer's disease by taking advantage of smartphones to collect multiple streams of behavioral information including active data such as reaction/response time to cognitive tasks as well as passive data such as mobility and sleep patterns. This is a really great idea as the diagnosis of AD is challenging and often takes several years before a provider can do so with confidence. It is likely that observing patterns in the home will expedite this diagnosis process.

The methods proposed herein are very interesting. I like the overall design and approach. My only concern is the sample size. That said, they need to pilot this to get started, so this did not diminish my score too much.

Suggested revisions to aims (if applicable):

Specific aim 1: Creation and refinement of the Smartphone Monitoring Assessment in Real Time – Alzheimer (SMART-A). This involves the development of popup cognitive tasks within our platform mindlamp2 to capture active and passive data. We will continue to refine this throughout the course of the project.

Specific aim 2: To identify the ecological data that best detect and track cognitive impairment in Alzheimer's disease patients.

Sub-aim 2A: To identify the ecological data that best identifies cognitive impairment cause by Alzheimer's disease. We will run our SMART-A app on 15 patients with Alzheimer's disease and 15 controls for 1 month to identify the combination of passive and active data that best differentiates the two groups.

Sub-aim 2B: To determine how this ecological data changes over time. We will run our SMART-A app on patients with Alzheimer's disease for multiple months to examine the day-to-day and month-to-month changes of their passive and active data.
