# Supplementary material for: Real-Time Smartphone Monitoring Assessments as a Cognitive Biomarker of Alzheimer Disease: Protocol for a Development Study
Source: JMIR Res Protoc. 2026 Jul 3;15:e93259. doi: 10.2196/93259 (PMC13331333; doi:10.2196/93259)
Supplement: Peer Review Report 2 [file resprot-v15-e93259-s002.pdf]

Mobile technology ...

Applicant Name
Chun Lim

Applicant Email
clim@bidmc.harvard.edu

Applying for:
Third Annual a2 Pilot Awards
created: Jun 30, 2023

YOUR OTHER APPLICATIONS

Coordinating Center Admin
December 15, 2023 at 1:47 PM

PennAITech Round 2 Application Feedback:

Reviewer 1: Significance: The project is highly significant due to low rates of detection of cognitive impairment and dementia among older adults and the need for feasible and acceptable approaches to increase detection.

Innovation: The project has several sources of innovation including the use of both passive and active data streams and the use of machine learning to identify markers of cognitive impairment.

Commercialization potential: Potential is high due to the use of the mindlamp application platform which could be a dapted by other health systems.

Approach: The approach is appropriate and the methods are described in detail. For both Aims 1 and 2A, the hypot heses state that the proposed approach will "improve patient compliance" and "identify Alzheimer's disease patient s with greater precision"; the comparison group for testing these hypotheses is unclear.

Investigators: The investigators have the skills and experience needed to complete the project.

Environment: The environment is outstanding and the project builds on existing BIDMC resources and work in this a rea.

Reviewer 2: This project aims to develop a smartphone-based app ('SMART-A') to capture data on AD patients, wit h the ultimately goal of being able to identify and track AD patients. The current project builds on a platform develo ped by the researchers for smartphone data collection and research, and aims to adapt and test it for the AD conte xt. While the overall goal of the project is exciting and the researchers have experience in this domain, enthusiasm i s dampened by weaknesses in the Approach.

Aim 1: There is a lot of description of the existing platform and its capabilities, but less information on what is actual ly being done in this Aim for the novel SMART-A platform, and how this will be done. Two sentences describe what will be adapted: presumably survey and memory questions as well as cognitive-based tasks. One notable strength here is the researcher's attention to digital health engagement, which is an important (and oft-ignored) feature for d ownstream usability.

Aim 2: This Aim will identify the data that best detects and tracks cognitive impairment across two sub-aims. In Aim 2A, 15 AD and 15 controls will use the platform; in Aim 2B, a longitudinal study will be conducted with a subset of th e AD patients that will also involve periodic in-clinic visits to assess cognitive and functional performance. This Aim lacked sufficient detail on study procedures and justification: why were 15 patients selected (for each group) and wi ll a month of data collection be sufficient to identify differences? How will healthy patients be recruited?

It is stated that subjects without smartphones will be loaned a smartphone in lieu of payment. While the gesture to wards equity is recognized, the details need clarification ' why is payment not provided to these individuals? One of the most significant issues with the provision of a second device in these types of studies is that they hardly get us ed (especially if subjects are accustomed to using a separate device for calls) thus there may be little passive data collection. This is especially a concern given that the smartphone will only be provided for a month. How will resear chers ensure that subjects actually use the second device?
